# 4-(1*H*-Tetra­zol-5-yl)pyridinium chloride

**DOI:** 10.1107/S1600536810047756

**Published:** 2010-11-24

**Authors:** Yan-Wei Zhang

**Affiliations:** a Department of Chemical & Environmental Engineering, Anyang Institute of Technology, Anyang 455000, People’s Republic of China

## Abstract

In the cation of the title compound, C_6_H_6_N_5_^+^·Cl^−^, the tetra­zole and pyridine rings are nearly coplanar, making a dihedral angle of 5.58 (11)°. The organic cations are linked to the chloride anions *via* N—H⋯Cl hydrogen bonds, forming chains along [110].

## Related literature

For supra­molecular self-assembly chemistry, see: Fender *et al.* (2002 ▶). For the structures of related tetra­zole derivatives, see: Fu *et al.* (2009 ▶).
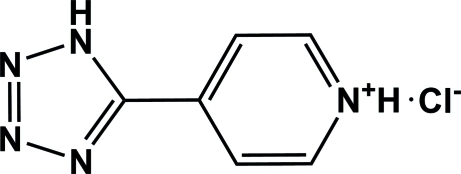


## Experimental

### 

#### Crystal data

C_6_H_6_N_5_^+^·Cl^−^
*M* *_r_* = 183.61
Monoclinic, 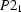

*a* = 4.8552 (10) Å
*b* = 7.5862 (15) Å
*c* = 10.884 (2) Å
β = 92.88 (3)°
*V* = 400.36 (14) Å^3^
*Z* = 2
Mo *K*α radiation
μ = 0.42 mm^−1^
*T* = 298 K
0.30 × 0.05 × 0.05 mm

#### Data collection

Rigaku Mercury CCD diffractometer
Absorption correction: multi-scan (*CrystalClear*; Rigaku, 2005 ▶) *T*_min_ = 0.910, *T*_max_ = 1.000
4104 measured reflections
1825 independent reflections
1687 reflections with *I* > 2σ(*I*)
*R*_int_ = 0.024

#### Refinement

*R*[*F* ^2^ > 2σ(*F* ^2^)] = 0.031
*wR*(*F* ^2^) = 0.071
*S* = 1.11
1825 reflections
109 parameters
1 restraint
H-atom parameters constrained
Δρ_max_ = 0.16 e Å^−3^
Δρ_min_ = −0.26 e Å^−3^
Absolute structure: Flack (1983 ▶), 840 Friedel pairs
Flack parameter: 0.07 (6)

### 

Data collection: *CrystalClear* (Rigaku, 2005 ▶); cell refinement: *CrystalClear*; data reduction: *CrystalClear*; program(s) used to solve structure: *SHELXTL* (Sheldrick, 2008 ▶); program(s) used to refine structure: *SHELXTL*; molecular graphics: *SHELXTL*; software used to prepare material for publication: *SHELXTL*.

## Supplementary Material

Crystal structure: contains datablocks I, global. DOI: 10.1107/S1600536810047756/xu5091sup1.cif

Structure factors: contains datablocks I. DOI: 10.1107/S1600536810047756/xu5091Isup2.hkl

Additional supplementary materials:  crystallographic information; 3D view; checkCIF report

## Figures and Tables

**Table 1:** Hydrogen-bond geometry (Å, °)

*D*—H⋯*A*	*D*—H	H⋯*A*	*D*⋯*A*	*D*—H⋯*A*
N1—H1*A*⋯Cl1^i^	0.86	2.21	3.0704 (18)	176
N5—H5*A*⋯Cl1^ii^	0.86	2.22	3.0344 (18)	159

Symmetry codes: (i) 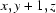; (ii) 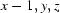.

**Fig. 1.**
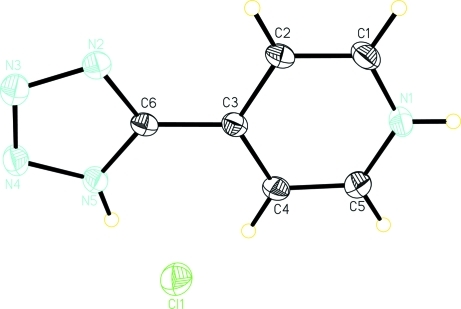
A view of the title compound with the atomic numbering scheme. Displacement ellipsoids were drawn at the 30% probability level.

**Fig. 2.**
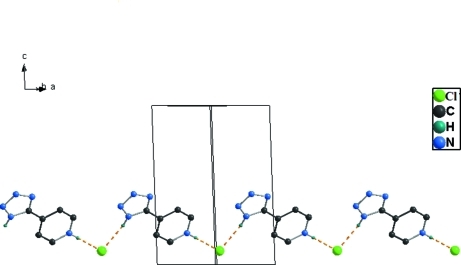
Part of the crystal packing of the title compound. H atoms not involved in hydrogen bonding (dashed lines) have been omitted for clarity.

## supplementary crystallographic information

### Comment

In recent years there is a rapidly increasing interest in the construction of various kinds of supramolecular systems for understanding molecular self-assembly principles and for designing molecular recognition devices (Fender *et al.* 2002). We report here the crystal structure of the title compound, 4-(1*H*-tetrazol-5-yl)pyridinium chloride.

In the title compound (Fig.1), the pyridine N atom is protonated. The tetrazole and pyridine rings are nearly coplanar and only twisted from each other by a dihedral angle of 5.58 (11)°. The geometric parameters of the tetrazole rings are comparable to those in related molecules (Fu *et al.*, 2009).

In the crystal structure, the organic cations are connected by the Cl^-^ anions through two type of N—H···Cl hydrogen bonds, with the N···Cl distance of 3.0704 (2)Å and 3.0344 (2) Å, respectively. Those H-bonds link the ion units into a one-dimensional chain along the [1 1 0] direction (Table 1 and Fig. 2).

### Experimental

4-(1H-Tetrazol-5-yl)pyridinium chloride was obtained commercially, and the single crystals were obtained from an ethanol solution.

### Refinement

H atoms attached to N atoms were located in a difference Fourier map, and refined in riding mode with N–H = 0.86 Å and U_iso_(H) = 1.2U_eq_(N). Other H atoms were fixed geometrically and treated as riding with C–H = 0.93 Å and U_iso_(H) = 1.2U_eq_(C).

### Crystal data

**Table:**

C_6_H_6_N_5_^+^·Cl^−^	*F*(000) = 188
*M**_r_* = 183.61	*D*_x_ = 1.523 Mg m^−^^3^
Monoclinic, *P*2_1_	Mo *K*α radiation, λ = 0.71073 Å
Hall symbol: P 2yb	Cell parameters from 1825 reflections
*a* = 4.8552 (10) Å	θ = 3.3–27.5°
*b* = 7.5862 (15) Å	µ = 0.42 mm^−^^1^
*c* = 10.884 (2) Å	*T* = 298 K
β = 92.88 (3)°	Block, colorless
*V* = 400.36 (14) Å^3^	0.30 × 0.05 × 0.05 mm
*Z* = 2	

### Data collection

**Table:**

Rigaku Mercury CCD diffractometer	1825 independent reflections
Radiation source: fine-focus sealed tube	1687 reflections with *I* > 2σ(*I*)
graphite	*R*_int_ = 0.024
Detector resolution: 13.6612 pixels mm^-1^	θ_max_ = 27.5°, θ_min_ = 3.3°
φ and ω scan	*h* = −6→6
Absorption correction: multi-scan (*CrystalClear*; Rigaku, 2005)	*k* = −9→9
*T*_min_ = 0.910, *T*_max_ = 1.000	*l* = −14→14
4104 measured reflections	

### Refinement

**Table:**

Refinement on *F*^2^	Secondary atom site location: difference Fourier map
Least-squares matrix: full	Hydrogen site location: inferred from neighbouring sites
*R*[*F*^2^ > 2σ(*F*^2^)] = 0.031	H-atom parameters constrained
*wR*(*F*^2^) = 0.071	*w* = 1/[σ^2^(*F*_o_^2^) + (0.029*P*)^2^ + 0.0441*P*] where *P* = (*F*_o_^2^ + 2*F*_c_^2^)/3
*S* = 1.11	(Δ/σ)_max_ < 0.001
1825 reflections	Δρ_max_ = 0.16 e Å^−^^3^
109 parameters	Δρ_min_ = −0.26 e Å^−^^3^
1 restraint	Absolute structure: Flack (1983), 840 Friedel pairs
Primary atom site location: structure-invariant direct methods	Flack parameter: 0.07 (6)

### Special details

**Table:**

Geometry. All e.s.d.'s (except the e.s.d. in the dihedral angle between two l.s. planes) are estimated using the full covariance matrix. The cell e.s.d.'s are taken into account individually in the estimation of e.s.d.'s in distances, angles and torsion angles; correlations between e.s.d.'s in cell parameters are only used when they are defined by crystal symmetry. An approximate (isotropic) treatment of cell e.s.d.'s is used for estimating e.s.d.'s involving l.s. planes.
Refinement. Refinement of *F*^2^ against ALL reflections. The weighted *R*-factor *wR* and goodness of fit *S* are based on *F*^2^, conventional *R*-factors *R* are based on *F*, with *F* set to zero for negative *F*^2^. The threshold expression of *F*^2^ > σ(*F*^2^) is used only for calculating *R*-factors(gt) *etc*. and is not relevant to the choice of reflections for refinement. *R*-factors based on *F*^2^ are statistically about twice as large as those based on *F*, and *R*- factors based on ALL data will be even larger.

### Fractional atomic coordinates and isotropic or equivalent isotropic displacement parameters (Å^2^)

**Table:**

	*x*	*y*	*z*	*U*_iso_*/*U*_eq_	
N3	0.6191 (4)	0.2346 (2)	0.94401 (17)	0.0448 (5)	
C4	0.5024 (4)	0.7071 (3)	0.67942 (18)	0.0354 (4)	
H4	0.3611	0.6417	0.6407	0.043*	
N4	0.4093 (4)	0.2114 (2)	0.86534 (18)	0.0426 (4)	
N5	0.3900 (3)	0.3586 (2)	0.79785 (15)	0.0356 (4)	
H5A	0.2678	0.3786	0.7396	0.043*	
C6	0.5897 (4)	0.4690 (2)	0.83517 (16)	0.0290 (4)	
N1	0.7754 (3)	0.9626 (2)	0.69060 (16)	0.0390 (4)	
H1A	0.8175	1.0632	0.6604	0.047*	
C2	0.8551 (4)	0.7442 (3)	0.83953 (19)	0.0360 (5)	
H2	0.9528	0.7030	0.9094	0.043*	
N2	0.7348 (4)	0.3944 (2)	0.92686 (16)	0.0389 (4)	
C1	0.9159 (5)	0.9050 (3)	0.7917 (2)	0.0400 (5)	
H1	1.0541	0.9743	0.8292	0.048*	
C5	0.5724 (4)	0.8690 (3)	0.63512 (18)	0.0401 (5)	
H5	0.4775	0.9142	0.5657	0.048*	
C3	0.6467 (4)	0.6419 (3)	0.78358 (16)	0.0293 (4)	
Cl1	0.95294 (9)	0.32109 (6)	0.59025 (4)	0.04239 (15)	

### Atomic displacement parameters (Å^2^)

**Table:**

	*U*^11^	*U*^22^	*U*^33^	*U*^12^	*U*^13^	*U*^23^
N3	0.0493 (10)	0.0393 (10)	0.0450 (10)	−0.0036 (9)	−0.0050 (8)	0.0062 (9)
C4	0.0335 (10)	0.0399 (11)	0.0322 (10)	−0.0090 (9)	−0.0049 (8)	−0.0024 (8)
N4	0.0449 (10)	0.0337 (10)	0.0484 (10)	−0.0080 (8)	−0.0059 (8)	0.0056 (8)
N5	0.0347 (8)	0.0346 (11)	0.0366 (8)	−0.0080 (7)	−0.0062 (7)	0.0042 (7)
C6	0.0258 (9)	0.0326 (10)	0.0286 (8)	−0.0027 (7)	−0.0009 (7)	−0.0047 (7)
N1	0.0460 (10)	0.0302 (9)	0.0412 (9)	−0.0088 (8)	0.0058 (8)	0.0008 (7)
C2	0.0338 (10)	0.0387 (11)	0.0345 (10)	−0.0071 (9)	−0.0083 (8)	−0.0026 (9)
N2	0.0416 (9)	0.0362 (9)	0.0377 (9)	−0.0024 (8)	−0.0082 (7)	0.0023 (7)
C1	0.0381 (11)	0.0380 (11)	0.0436 (12)	−0.0112 (9)	−0.0019 (9)	−0.0067 (10)
C5	0.0442 (11)	0.0419 (13)	0.0338 (10)	−0.0059 (9)	−0.0021 (9)	0.0036 (8)
C3	0.0296 (9)	0.0299 (9)	0.0285 (9)	−0.0029 (7)	0.0028 (7)	−0.0042 (7)
Cl1	0.0465 (3)	0.0433 (3)	0.0362 (2)	−0.0112 (3)	−0.00936 (18)	0.0070 (2)

### Geometric parameters (Å, °)

**Table:**

N3—N4	1.309 (3)	C6—C3	1.459 (3)
N3—N2	1.353 (2)	N1—C5	1.334 (3)
C4—C5	1.369 (3)	N1—C1	1.338 (3)
C4—C3	1.393 (3)	N1—H1A	0.8600
C4—H4	0.9300	C2—C1	1.365 (3)
N4—N5	1.337 (2)	C2—C3	1.391 (3)
N5—C6	1.330 (2)	C2—H2	0.9300
N5—H5A	0.8600	C1—H1	0.9300
C6—N2	1.320 (2)	C5—H5	0.9300
			
N4—N3—N2	110.18 (18)	C1—C2—C3	119.9 (2)
C5—C4—C3	118.78 (19)	C1—C2—H2	120.0
C5—C4—H4	120.6	C3—C2—H2	120.0
C3—C4—H4	120.6	C6—N2—N3	106.21 (16)
N3—N4—N5	106.13 (17)	N1—C1—C2	119.6 (2)
C6—N5—N4	109.17 (15)	N1—C1—H1	120.2
C6—N5—H5A	125.4	C2—C1—H1	120.2
N4—N5—H5A	125.4	N1—C5—C4	120.6 (2)
N2—C6—N5	108.31 (17)	N1—C5—H5	119.7
N2—C6—C3	124.90 (17)	C4—C5—H5	119.7
N5—C6—C3	126.77 (16)	C2—C3—C4	118.91 (18)
C5—N1—C1	122.22 (18)	C2—C3—C6	118.76 (17)
C5—N1—H1A	118.9	C4—C3—C6	122.33 (17)
C1—N1—H1A	118.9		

### Hydrogen-bond geometry (Å, °)

**Table:**

*D*—H···*A*	*D*—H	H···*A*	*D*···*A*	*D*—H···*A*
N1—H1A···Cl1^i^	0.86	2.21	3.0704 (18)	176
N5—H5A···Cl1^ii^	0.86	2.22	3.0344 (18)	159

Symmetry codes: (i) *x*, *y*+1, *z*; (ii) *x*−1, *y*, *z*.

## References

[bb1] Fender, N. S., Kahwa, I. A. & Fronczek, F. R. (2002). *J. Solid State Chem.* **163**, 286–293.

[bb2] Flack, H. D. (1983). *Acta Cryst.* A**39**, 876–881.

[bb3] Fu, D.-W., Ge, J.-Z., Dai, J., Ye, H.-Y. & Qu, Z.-R. (2009). *Inorg. Chem. Commun.* **12**, 994–997.

[bb4] Rigaku (2005). *CrystalClear* Rigaku Corporation, Tokyo, Japan.

[bb5] Sheldrick, G. M. (2008). *Acta Cryst.* A**64**, 112–122. 10.1107/S0108767307043930 18156677

